# Autologous mesenchymal stem cell application for cartilage defect in recurrent patellar dislocation: A case report

**DOI:** 10.1016/j.ijscr.2019.01.031

**Published:** 2019-01-31

**Authors:** Andri Maruli Tua Lubis, Troydimas Panjaitan, Charles Hoo

**Affiliations:** a Department of Orthopaedics and Traumatology, Faculty of Medicine Universitas Indonesia/Cipto Mangunkusumo Hospital, Diponegoro 71, Jakarta, Indonesia; b Mitra Keluarga Kelapa Gading, Jakarta, Indonesia; c Mayapada Hospital, Jakarta, Indonesia

**Keywords:** Autologous mesenchymal stem cells, Cartilage defect, Recurrent patellar dislocation, Case report

## Abstract

**Introduction:**

Recurrent patellar dislocation can lead to articular cartilage injury. We report a 21-year old male with left patella instability and articular cartilage defect.

**Presentation of case:**

A 21-year-old male presented with left patellar instability and pain. Knee range of motion (ROM) was limited when patella was dislocated (0–20°). The J-sign positive, patellar apprehension test was positive, with medial patella elasticity/patellar glide >2 quadrants. The Q angle, in the 90° flexed knee position was still normal. The plain radiograph imaging showed no abnormality. Insall-Salvati index was 1.12. The patient was diagnosed with recurrent patellar dislocation and cartilage lesion of the left knee, and was treated with combining Fulkerson osteotomy with the lateral retinacular release and percutaneous medial plication, followed by microfracture procedure and MSCs implantation.

**Discussion:**

Recurrent patellar dislocation is uncommon problem while cartilage lesions following recurrent patellar dislocations are quite common, but still no consensus on the management.

**Conclusion:**

Combination of Fulkerson osteotomy with the lateral retinacular release and percutaneous medial plication was effective in treating chronic patellar instability. The microfracture procedure and MSCs implantation was safe and could improve the cartilage regeneration in patients with articular cartilage defect due to recurrent patellar dislocation.

## Highlights

• This is a novel management of a 21-year-old male with recurrent patellar dislocation.
• Fulkerson procedure was to realign the patella.
• Mesenchymal stem cell application was used for the articular cartilage defect.
• The outcome of this combination treatment was satisfying.

## 1 Introduction

Recurrent patellar dislocation is a repeated dislocation that follows from an initial episode of minor trauma dislocation [1]. Conservative management gives a minimal result in re-dislocation, with persistent symptoms of anterior knee pain, instability and activity limitation. Meanwhile, there is no gold standard treatment of realignment procedures [[2], [3], [4]]. This can further cause cartilage lesion in the patella and femoral condyle, and consequently increase the risk of re-dislocation [5,6]. Mesenchymal stem cells (MSCs) have been widely explored for treating cartilage defect due to their potency of chondrogenic differentiation [[7], [8], [9]]. We present a novel approach of treating cartilage lesions in recurrent patellar dislocation by combining of arthroscopic microfracture and autologous bone marrow derived MSCs (BM-MSCs) after Fulkerson osteotomy.

This work has been reported in line with the SCARE criteria [10].

## 2 Presentation of case

A 21-year-old male presented with left knee discomfort. Ten years ago, the patient felt discomfort on the medial side of the knee and felt his knee cap slide out laterally. The patient experienced several episodes of instability ranging from a feeling of “giving away” until a prominent lateral sliding-off of his knee cap. Anterior knee pain has also occurred during activities such as climbing stairs or exercising.

Physical examination revealed slight pain on the anterior side of the patella, but no atrophy or squinting patella. Knee range of motion (ROM) was normal when the knee cap position was normal, but was limited when it was dislocated (0–20°). Lateral subluxation of the patella was found when the knee was extended from 90° flexion position (J-sign positive), positive patellar apprehension test, with medial patella elasticity/patellar glide >2 quadrants. The Q angle, in the 90° flexed knee position, was 10°, which was still normal. The plain radiograph imaging showed no abnormality. Insall-Salvati index was 1.12 [11]. The patient was diagnosed with recurrent patellar dislocation, with suspected cartilage lesion of the left knee.

**Fig. 1:**
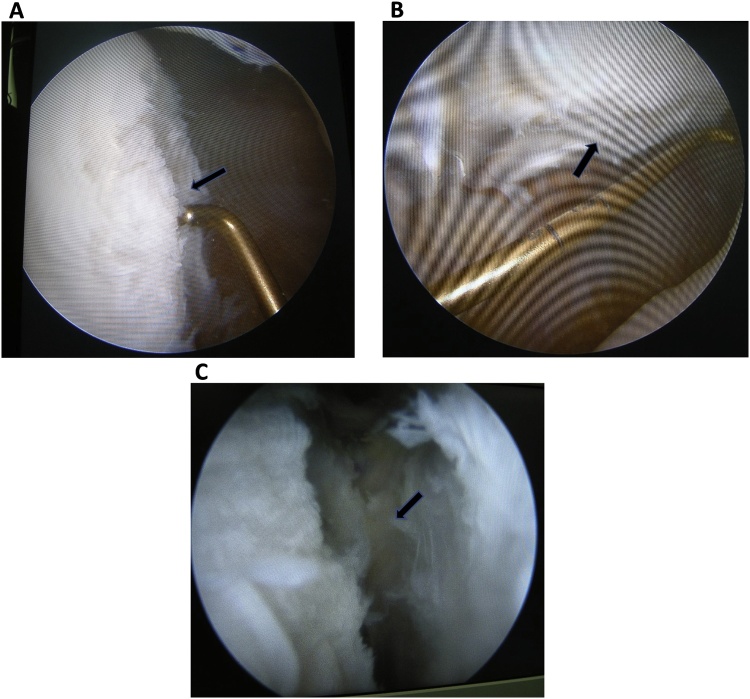
**A**. Cartilage defect on the femoral lateral condyle with a diameter of 3 cm (pointed by the arrow). **B**. Articular cartilage defect on posteromedial patella with a diameter of 2.5 cm (pointed by the arrow). **C**. Lateral retinaculum dissection/lateral release using an electrocautery (pointed by the arrow).

**Fig. 2:**
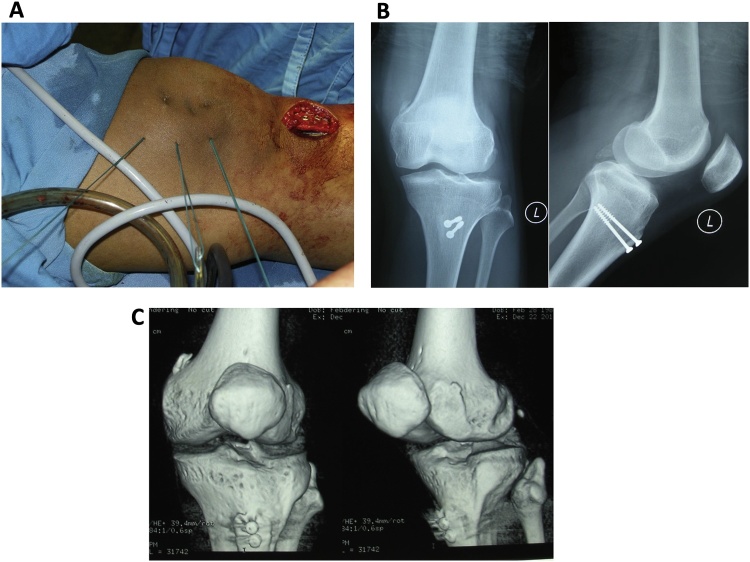
**A**. Percutaenous medial plication using non-absorbable string no.2. **B**. Post-operative anteroposterior and lateral projection of plain radiograph imaging. **C**. Post-operative CT scan.

The first surgery was an arthroscopy diagnostic and distal realignment procedure (lateral retinaculum release, percutaneous medial retinaculum plication, and antero-medialization of tibia tubercle/Fulkerson osteotomy). We found articular cartilage defects on the lateral condyle of the femur with a diameter of 3 cm (Fig. 1A), and on the postero-medial with a diameter of 2.5 cm (Fig. 1B), and the depth of both was more than 50% of the cartilage thickness. We determined that the articular defect was Grade 3 according to International Cartilage Regeneration & Joint Preservation Society (ICRS) [12]. We performed a dissection of lateral retinaculum (lateral release) (Fig. 1C) using an electrocautery, continued by incising the medial side of tibia tuberosity and detaching the patellar tendon by using an oblique osteotomy procedure on tibia tuberosity, where the fragment slide 1 cm antero-medially and fixed with two 3.5 mm (length 40 mm) partial threaded cancellous screw, followed by percutaneous plication on the medial side of the patella using non-absorbable string (Fig. 2A). Post-operative ROM was 90° flexion without any dislocation (Fig. 2B) and the position of the screws was good (Fig. 2C).

**Fig. 3:**
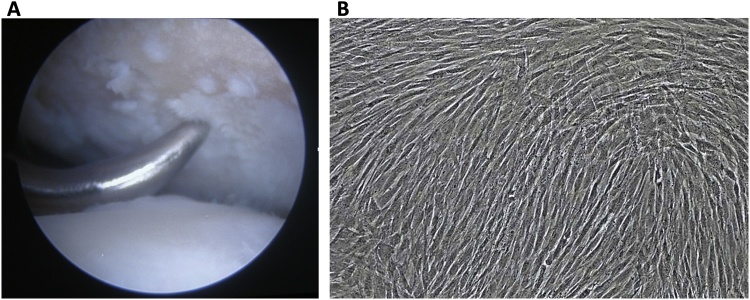
**A**. Arthroscopic microfracture on cartilage defect using an awl, with a depth of ±4 cm. **B**. The mesenchymal stem cell culture after day 22 showing fibroblast-like cell/spindle shaped cells that 100% confluent.

One month after surgery, full ROM and weight bearing exercises were started, including knee exercise until maximum flexion was reached along with quadriceps muscle exercise. Eighteen month after that surgery, we performed an iliac crest bone marrow aspiration; arthroscopic microfracture by using an awl until 4 mm depth was reached on the site located ±3–4 mm from the articular cartilage defect on the posteromedial patella and femoral lateral condyle (Fig. 3A); and tibial tuberosity screw removal.

Approximately 30 mL of bone marrow was aspirated from the posterior iliac crest. Bone marrow aspirate was diluted in phosphate-buffered saline (PBS) and centrifuged at room temperature. The buffy coat was washed and cultivated for 3–4 weeks until reaching the required amount (10^7^ MSCs/mL) (Fig. 3B). The cells were harvested and characterized with flow cytometer. The MSCs, having negative bacteria and fungi tests, were injected intra-articularly into the left knee. Then, 2 mL HA were injected weekly for 3 weeks. Non-weight bearing exercise was conducted for 6 weeks.

**Fig. 4:**
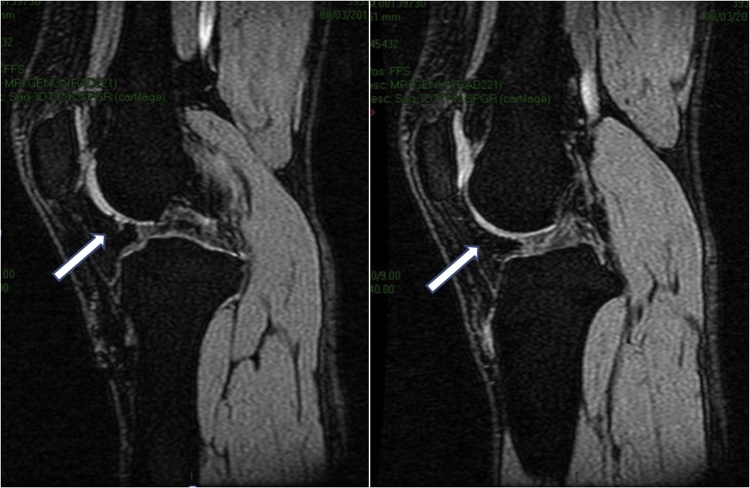
MRI FSE cor T2-weighted signal in different slice. Left image showing cartilage defect (pointed by the arrow). Right image showing cartilage growth was found in the defect (pointed by the arrow).

Outcomes were assessed by using International Knee Documentation Committee (IKDC) score, visual analog scale (VAS) score and imaging. Baseline IKDC score was 52.9 and VAS score was 8. Nineteen months after the first surgery, IKDC score was improved to 93.1, while the VAS score decreased to 2. Six months after MSCs implantation, evaluation by MRI FSE cor T2-weighted signal (cartilage sequence) showed a significant growth of articular cartilage covering most of the defect (Fig. 4). Two years after the MSCs implantation, there was no complaint and full ROM was reached.

## 3 Discussion

Recurrent patellar dislocation are uncommon problem, with recurrence rate 15%–44% after conservative management [2], while cartilage lesions following recurrent patellar dislocations are quite common [5], but still no gold standard or consensus on the management [1,3]. This patient was diagnosed as chondromalacia Grade 3 Outerbridge classification and Grade 3 ICRS [5,12]. One of the suitable procedures for recurrent patellar dislocation with chondromalacia, especially Grade 3 or 4, was Oblique Fulkerson-type osteotomy, with or without the release of lateral retinaculum [13]. This distal realignment procedure could decrease patellofemoral pain by anteriorization of tibial tuberosity, decreasing articular contact pressure and at the same time medializing knee extensor mechanism [13,14]. Therefore, we performed the Fulkerson-type osteotomy with lateral retinacular release, combined with percutaneous medial plication since the patient was already 21 years of age and the bone was expected to be mature so that the risk of premature physeal closure in proximal tibia can be avoided [4]. This technique has demonstrated good results (86%), although it had a risk of tibial stress fracture in the healing process [4]. The lateral retinacular release is an adjuvant after tibial tubercle medialization to re-center the patella [1,14]. It was reported that isolated lateral retinacular release significantly gives an inferior long-term result compared to medial reefing [15]. Percutaneous plication of medial patella procedure was indicated to build a strong construct by shortening the patellofemoral ligament, in order to prevent lateral sliding of the patella [13].

Treatment of articular cartilage defect remains challenging since it has limited self-healing capacity. Lesions that do not reach the subchondral zone will be unlikely to heal and usually progress to a cartilage degeneration [16,17]. Limited blood supply in the cartilage and low chondrocyte metabolic activity disrupt natural healing that is supposed to fill the defect by increasing hyaline cartilage synthesis activity or stem cell mobilization from bone marrow to site of injury [16]. The proper initial procedure for chondral lesion >4 cm^2^ was marrow stimulation by mosaicplasty or microfracture; and for a lesion <4 cm^2^ and >12 cm^2^ accompanied with symptoms, autologous cartilage implantation (ACI) beneath a sutured periosteal flap was promising. This procedure could not regenerate cartilage in the long term, due to loss of flap or cell suspensions. A scaffold (e.g. HA) was then used to act as an anchorage for chondrocytes adherence on cartilage defects and to promote the secretion of chondrocyte extracellular matrix [16,17]. The BM-MSCs implantation could be an alternative source of the chondrocytes. Human BM-MSCs are relatively easy to isolate and to be cultured in such a condition that may retain their capability to differentiate into chondrocytes [9,16].

The MSCs effect was reported as effective as ACI and even had the advantage over ACI in terms of the number cells obtained, better proliferation capacity and less damage in the donor site [9,16]. Treating large cartilage defects by using BM-MSCs showed good outcome, but the transplantation procedure was invasive [18]. Wong et al. conducted a clinical study of the BM-MSCs intra-articular injection in combination with high tibial osteotomy (HTO) and microfracture for treating cartilage defect with varus knee. They reported that intra-articular MSCs injection improved the outcomes in the patients undergoing HTO and microfracture [19]. Here we performed also a less invasive approach by injecting the autologous BM-MSCs intra-articularly, following the arthroscopic microfracture using an awl to penetrate the subchondral bone plate in the cartilage defects, which led to clot formation. This clot contains progenitor cells, cytokines, growth factors and pluripotent, marrow-derived mesenchymal stem cells, which produce a fibrocartilage repair with varying amounts of type-II collagen content [20]. Cytokine within the fibrin clot will attract the injectable stem cells to the cartilage lesions.

The HA injection in this patient was aimed to suspend the MSCs and to support regenerative potency of MSCs with chondroinductive and chondroprotective potency of HA. Intraarticular injection of MSCs suspended in HA could be an alternative treatment for large cartilage defect [8]. Supporting microfracture technique by intra-articular HA injections had a positive effect on the repair tissue formation within the chondral defect [21,22]. The MRI showed that there was a growth of articular cartilage covering most of the defect even though it was not perfect as yet.

## 4 Conclusion

This case report demonstrated that combining Fulkerson osteotomy with the lateral retinacular release and percutaneous medial plication was effective in treating chronic patellar instability. The combination of microfracture and MSCs implantation was safe and could regenerate the articular cartilage in this patient.

## Conflicts of interest

Andri Lubis is a consultant for Conmed Linvatec and Pfizer Indonesia.

## Sources of funding

No sponsorship for this case report.

## Ethical approval

This is a case report; therefore it did not require ethical approval from ethics committee. However, we have got permission from the patient to publish his data.

## Consent

We have written and signed informed consent obtained from the patient to publish this case report and accompanying images.

## Author’s contribution

Andri Lubis contributed in performing the surgery and MSCs implantation, data collection, data analysis.

Troydimas Panjaitan contributed in data collection and data analysis.

Charles Hoo contributed in writing the paper.

## Registration of research studies

This is a case report, not a clinical study.

## Guarantor

The Guarantor is Andri M.T. Lubis, M.D., Ph.D.

## Provenance and peer review

Not commissioned, externally peer-reviewed.
